# Anthropogenic climate change will likely outpace coral range expansion

**DOI:** 10.1126/sciadv.adr2545

**Published:** 2025-06-06

**Authors:** Noam S. Vogt-Vincent, James M. Pringle, Christopher E. Cornwall, Lisa C. McManus

**Affiliations:** ^1^ Hawai’i Institute of Marine Biology, University of Hawai’i at Mānoa, Honolulu, HI, USA.; ^2^ Department of Earth Sciences, University of New Hampshire, Durham, NH, USA.; ^3^ School of Biological Sciences, Victoria University of Wellington, Wellington, New Zealand.

## Abstract

Past coral range expansions suggest that high-latitude environments may serve as refugia, potentially buffering coral biodiversity loss due to climate change. We explore this possibility for corals globally, using a dynamic metacommunity model incorporating temperature, photosynthetically available radiation, pH, and four distinct, interacting coral assemblages. This model reasonably reproduces the observed distribution and recent decline of corals across the Indo-Pacific and Caribbean. Our simulations suggest that there is a mismatch between the timescales of coral reef decline and range expansion under future predicted climate change. Whereas the most severe declines in coral cover will likely occur within 40 to 80 years, large-scale coral reef expansion requires centuries. The absence of large-scale coral refugia in the face of rapid anthropogenic climate change emphasizes the urgent need to reduce greenhouse gas emissions and mitigate nonthermal stressors for corals, both in the tropics and in higher latitudes.

## Teaser

Coral reef range expansion takes centuries, and higher-latitude seas are therefore ineffective refugia for most tropical corals.

## INTRODUCTION

Coral reefs are immeasurably valuable marine environments, supporting a third of marine species and the livelihoods of millions of people, particularly in low- and middle-income countries (*1*, *2*). Unfortunately, reef-building corals are also highly sensitive to environmental change, with the observed global decline in coral cover expected to continue under future climate projections (*3*).

As the ocean warms and isotherms migrate poleward, thermal niches shift to higher latitudes, and a rise in marine species diversity in subtropical and temperate seas is expected to accompany the decline in the tropics (*4*, *5*). This expected redistribution of marine life has led to the suggestion that high-latitude seas may act as climate change “refugia” for reef-building corals (*6*), potentially serving as a long-term buffer against declining coral diversity. The scope for latitudinal range expansion for corals may be limited by light attenuation, acidification, competition with macroalgae, local anthropogenic stressors, and a lack of settlement cues (*7*). Nevertheless, many statistical species distribution models support predictions of coral range expansion (*8*, *9*), and the geological record also provides evidence that coral reefs expand and contract in response to geologically recent environmental change (*10*–*13*). A rise in coral recruitment has already been observed at some subtropical field sites (*14*), and recent apparent poleward expansion has occurred in Australia, Florida, and Japan (*15*–*17*).

On the other hand, some of this recent range expansion may have been driven by the proliferation of coral species already present in high-latitude environments and taxonomic misidentification, rather than exclusively through the introduction of new species from the tropics (*18*–*20*), and these marginal communities are environmentally and functionally distinct from tropical coral reefs (*21*, *22*). Evidence of “topicalization” is conspicuously absent from other sites that should, in principle, be suitable targets for coral range expansion (*23*, *24*). The recent rate of warming is also exceptional within the context of the Late Pleistocene (*25*) and exceeds the rate that can be resolved from most geological records (*26*), making it challenging to contextualize the response of coral reefs to rapid paleoenvironmental change. It is therefore unclear how analogous or useful past coral range expansions are in predicting coral reef dynamics over the coming century. Corals are also relatively slow-growing organisms and are wholly reliant on passive larval dispersal to expand their range. As a result, the true distribution of corals will not necessarily follow habitat suitability if the rate of climate change exceeds their capacity to effectively colonize new environments. However, this is an assumption made by most species distribution models used to forecast coral range shifts.

The capacity for latitudinal coral range expansion therefore remains uncertain, limiting our ability to predict the impact of climate change on coral reef ecosystems. Range expansion has important implications for global coral distribution, diversity and ecosystem functioning. Furthermore, predicting where and when refugia for tropical corals—which we take here to refer to environments supporting the long-term persistence of tropical corals despite global climate change (*6*)—are likely to emerge may also permit proactive or dynamic management plans for environments that may not have previously been prioritized (*21*).

In this study, we expand on earlier work (*27*) to develop a simple process-based, eco-evolutionary metacommunity model, the Coral Eco-evolutionary Range Expansion Simulation (CERES), which reasonably represents the main processes that drive multidecadal coral reef dynamics. Using CERES, we demonstrate that there is a mismatch between the expected rate of future climate change and the rate of coral range expansion, indicating that range expansion alone is unlikely to buffer coral diversity against anthropogenic climate change.

## RESULTS

### Reproducing observed coral biogeography and trends

**Fig. 1.**
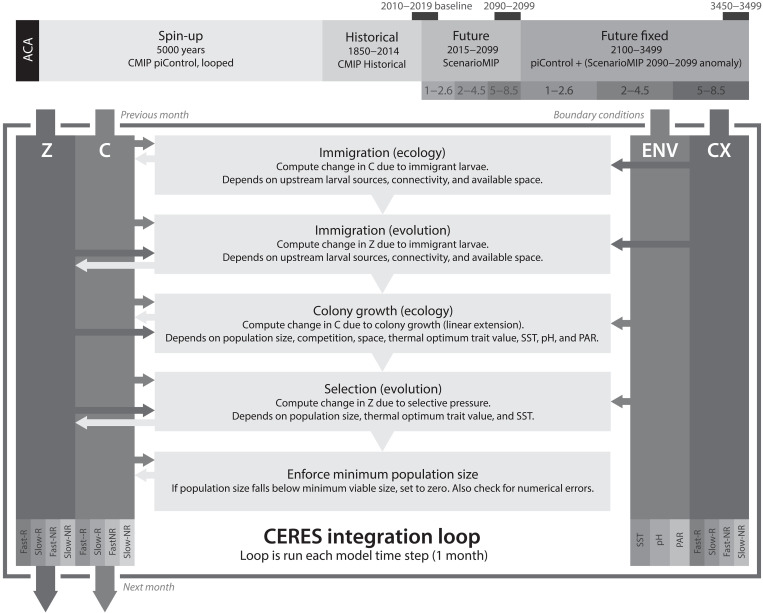
Experimental design (top) and schematic overview of the CERES integration loop (bottom). *Z* and *C* are the modeled dependent variables: the thermal optima and coral cover for each assemblage and subpopulation. “ENV” and “CX” represent the modeled independent variables: the environmental variables (SST, pH, and PAR) and potential connectivity. Fast/Slow-R/NR refer to coral assemblages (fast/slow-growing, reef/nonreef). Inward and outward arrows respectively represent variables being read and updated by the model.

CERES simulates the response of four hypothetical, interacting coral assemblages to environmental change using an eco-evolutionary metapopulation framework, assuming that populations grow through the linear extension of coral colonies and the establishment of new coral colonies through larval dispersal (Fig. 1, see also Materials and Methods). We do not explicitly model specific coral species; instead, these four assemblages are a useful abstraction that we use to qualitatively explore the response of coral reef environments to future climate change. We consider competing reef and nonreef assemblages, further divided into fast-growing and slow-growing life history strategies (*28*). CERES does not explicitly model the geological process of reef formation; as such, our reef assemblages instead represent the high-diversity coral communities that are characteristic of contemporary coral reefs, whereas nonreef assemblages represents coral communities characterized by lower diversity that are found outside true coral reefs. Multiple assemblages can coexist within sites, and a transition from reef to nonreef can be interpreted as coral reef degradation and diversity loss. Reef assemblages are parameterized by a narrower thermal tolerance, but are assumed to have a competitive advantage over nonreef assemblages in warmer environments. The fast-growing assemblages represent branching corals (primarily *Acropora*) with a greater growth rate and higher sensitivity to thermal stress, whereas the slow-growing assemblages largely represent domed and plating morphologies, particularly genera such as *Porites*, *Platygyra*, and *Montastraea*, characterized by a lower growth rate but higher tolerance to stress.

We assume that habitat shallower than 20 m is “potentially habitable” for corals, and simulate coral cover for each assemblage across 87,965 subpopulations (1/12° grid cells) at monthly temporal resolution. Colony linear extension rate is modeled as a function of the monthly sea surface temperature (SST), pH, photosynthetically available radiation (PAR) at the seafloor, and interassemblage competition; whereas population growth through the establishment of new colonies is based on estimates of fecundity, postspawning processes such as mortality, and potential connectivity (*29*). CERES further simulates a quantitative trait, the mean thermal optimum, for each assemblage and subpopulation. This trait value evolves based on immigration and selection, the latter being proportional to the additive genetic variance and the gradient of growth rate with respect to the trait value (*27*). The colony linear extension rate falls with distance from optimal environmental conditions, and assemblages experience mortality beyond low and high thermal stress thresholds (referenced to the thermal optimum) and under compound environmental stress. The growth rate dependence of each assemblage on pH, PAR, and competition does not evolve. We parameterize the model using empirical physiological measurements where possible, which we translate into a population growth rate by assuming that the size structure of coral colonies is log-normal (*30*) and static.

**Fig. 2.**
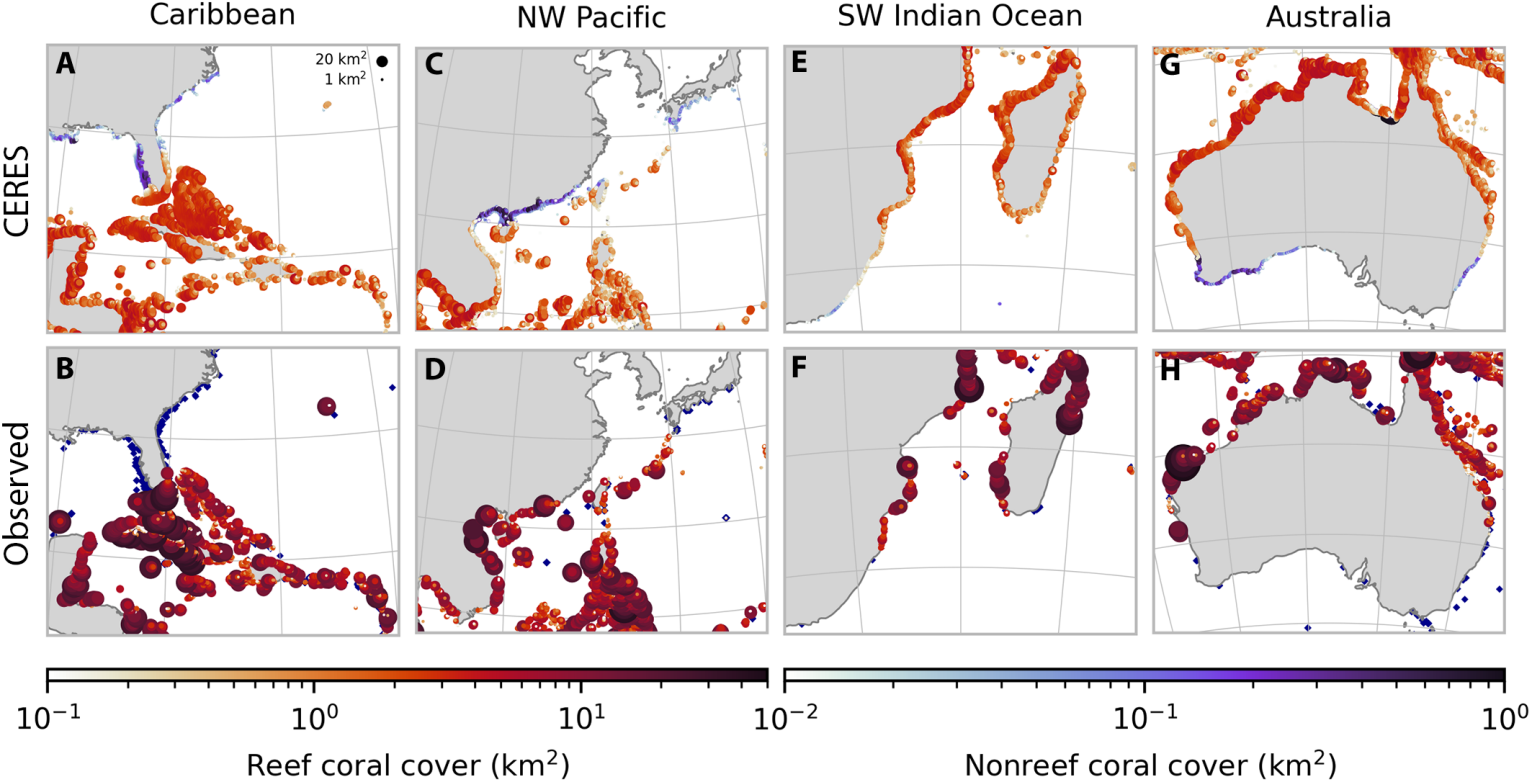
Comparison of simulated modern coral cover against observations. Marker colors represent the mean coral cover from 2010 to 2019 in CERES (top); and coral cover from the Allen Coral Atlas (*33*) and (as blue points) scleractinian coral occurrence records within 20 m depth (*98*) (bottom). Panels focus on the four major coral reef fronts in (**A** and **B**) the Caribbean, (**C** and **D**) NW Pacific, (**E** and **F**) SW Indian Ocean, and (**G** and **H**) Australia. To show both reef and nonreef coral cover from CERES, we only plot reef coral cover where it exceeds 0.1 km^2^. The Allen Coral Atlas does not distinguish between coral and algae on rocky substrate, so it may overestimate coral extent. See fig. S3 for a zoomed-in perspective and fig. S4 for a comparison with the UNEP-WCMC coral reef dataset (*99*).

Unlike statistical models, CERES is fully process based and predicts coral cover and thermal optima for each assemblage and subpopulation as emergent properties of the system. For each of the 12 Coupled Model Intercomparison Project Phase 6 (CMIP6) models (*31*) with a transient climate response in the “very likely” 1.2° to 2.4°C range (*32*), we impose a 5000-year spin-up period under repeating preindustrial forcing to allow corals to equilibrate to preindustrial conditions, followed by historical forcing from 1850 to 2014. Despite not being explicitly constrained by present-day species distributions, the emergent biogeography from the model resembles the true distribution of reef-building corals in the Indo-Pacific and NW Atlantic (Fig. 2 and fig. S1). The simulated latitudinal distribution and limit of reefal corals generally corresponds to the extent of coral reefs in the Allen Coral Atlas (*33*) within 100 to 200 km for the major coral reef fronts (figs. S2 to S4), although the latitudinal extent is underestimated in Japan, and potentially overestimated in other reef fronts. It is not unexpected that a simple process-based model with global parameterizations is unable to reproduce the regional distribution of coral reefs to the same accuracy as a statistical model. However, the broad consistency with observations across major reef-forming regions suggests that it is capturing the processes that set the distribution of coral reefs on a global scale. The “patchiness” of coral reefs tends to be underestimated by CERES as geomorphology and reef accretionary processes are not considered, resulting in some erroneous predictions such as high coral cover across the sandy Bahama Banks (Fig. 2, A and B) and overly continuous fringing reefs along continental coastlines. CERES overestimates the abundance of corals along eastern ocean boundaries, the Southwest Atlantic, and upwelling equatorial regions (fig. S1). These discrepancies are likely due to coral evolutionary history, and the effects of nutrients and/or sedimentation (see the Supplementary Materials). Because corals in these environments represent less than 1% of global coral cover (*34*), we exclude them from our analyses and focus on the Indo-Pacific and NW Atlantic.

CERES also successfully reproduces both regional and global declines in coral cover observed since the late 20th century by the Global Coral Reef Monitoring Network (GCRMN) (*34*), although it is on average slightly more conservative (fig. S5). For instance, the GCRMN estimated that global live hard coral cover declined by 3.2% in absolute terms between the 2005 to 2009 and 2015 to 2019 monitoring periods, compared to 2.0% (1.0 to 3.4% across the model ensemble) for CERES. CMIP models are not assimilative, so CERES would not be expected to reproduce the correct timing of global bleaching events, but CERES nevertheless simulates lower coral cover variability compared to observations. While records of recent coral range expansion are limited, coral community composition in Japan has been periodically monitored since the 1930s (*17*). It is unclear to what extent observed changes represent a genuine range shift as opposed to an expansion of existing high-latitude coral communities (*19*), but most CERES ensemble members predict an increase in coral cover at sites where coral communities may have expanded over recent decades (fig. S6), in agreement with observations (*17*). On the other hand, changes in coral density (both positive and negative) are lower in magnitude in CERES (fig. S7) compared to observations (*14*).

We emphasize that CERES is a simple dynamic model built for mechanistic understanding, rather than optimizing model-data fit. As such, we do not recommend overinterpreting any one specific model result, particularly at a regional level and subdecadal timescales. However, given that the model can independently (on the basis of empirical physiological data) reproduce coral reef distributions and decadal trends in coral cover, we consider that it reasonably represents the processes that govern multidecadal coral population dynamics, indicating that it is a useful model for exploring the potential response of coral reefs to future climate change.

### The future of coral reefs under anthropogenic climate change

**Fig. 3.**
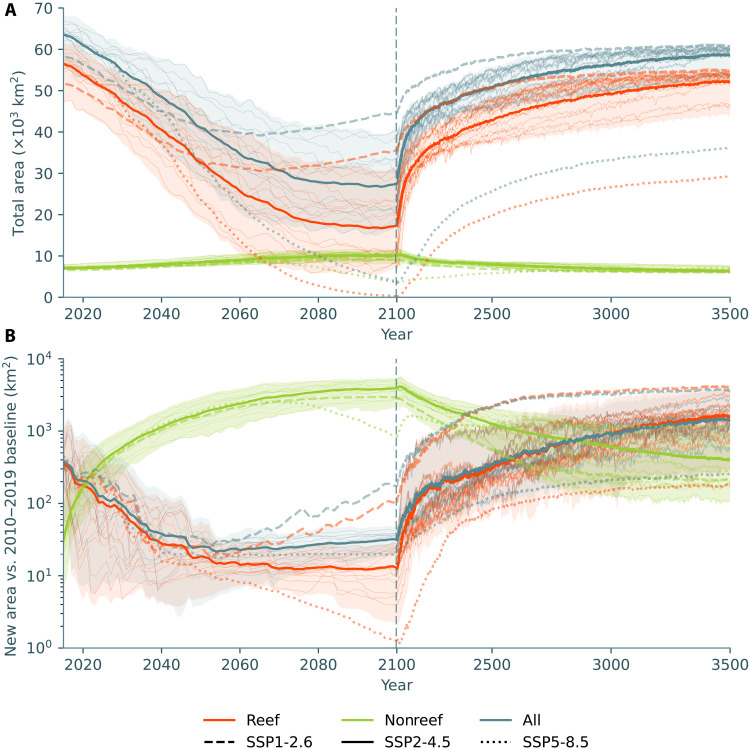
Global trends in simulated coral cover. (**A**) Total modeled area of all (blue), reef (orange), and nonreef (green) corals across the Indo-Pacific and NW Atlantic. Thick lines show the ensemble mean for SSP2-4.5 (solid), SSP1-2.6 (dashed), and SSP5-8.5 (dotted). Thin lines and the shaded area show trajectories for individual models for SSP2-4.5. (**B**) Total new coral cover relative to the mean model state from 2010 to 2019. New coral cover is high during the 2010 to 2019 baseline due to interannual variability. Note that the time axis scale changes in the year 2100, exaggerating the post-2100 recovery. Simulations after 2100 assume that SST and pH are static (apart from seasonal variability).

**Fig. 4.**
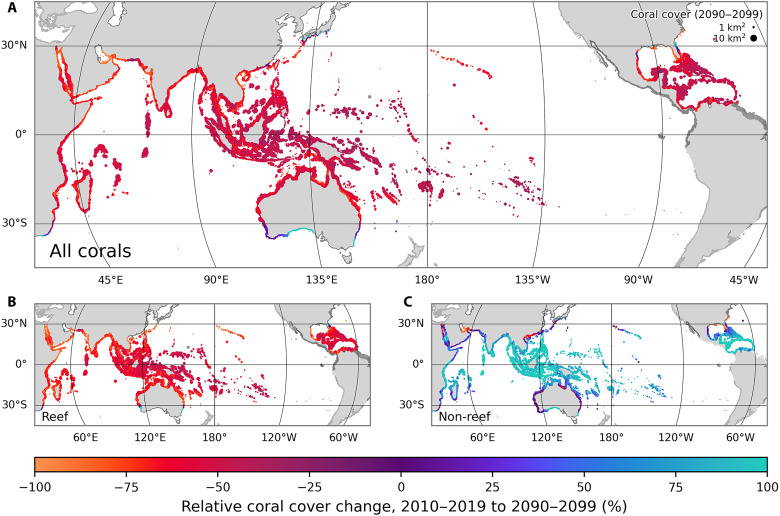
Spatial variability in simulated 21st-century coral cover change. Marker colors represent the mean relative change in coral cover from 2010-2019 to 2090-2099 under SSP2-4.5 across all ensemble members in CERES for (**A**) all, (**B**) reef, and (**C**) nonreef coral. Sites are scaled by the absolute coral cover between 2090 and 2099.

At the end of the CMIP6 historical period, we impose SST and pH from the “sustainable” SSP1-2.6, “middle of the road” SSP2-4.5, and “fossil-fueled development” SSP5-8.5 ScenarioMIP scenarios (*31*) until the end of the 21st century, again from 12 CMIP6 models. CERES predicts that coral cover declines by an average of 58% by the end of the 21st century relative to 2010 to 2019 under SSP2-4.5 (Fig. 3A). There is considerable variability in the predicted decline across the CMIP6 ensemble, ranging from 41 to 71%. Although there is geographic variability, this decline exceeds 25% at almost all locations (Fig. 4A and fig. S8).

The ensemble-mean coral cover decline of 58% masks the considerably higher decline of 71% (49 to 86% across the ensemble) predicted for coral reefs under SSP2-4.5 (Figs. 3A and 4B), with many present-day reefs experiencing near or complete collapse (fig. S8 and movie S1). In contrast, the model predicts that the spatial cover of nonreef coral communities will increase by 41% (24 to 59%) over the coming century (Fig. 4C). This increase is largely in the form of reefal assemblages transitioning into nonreefal assemblages (the model analog for the degradation of contemporary coral reefs into lower diversity coral communities) rather than the formation of new coral habitat. Under the sustainable SSP1-2.6 scenario, CERES predicts an average decline of 25% (11 to 45%) for coral cover, rising to 34% (15 to 59%) for reef corals. As expected, the fossil-fueled development SSP5-8.5 scenario results in a catastrophic 92% (85 to 99%) fall in coral cover and a virtual elimination (96 to 100%) of coral reefs.

In contrast to predictions from some statistical models, CERES predicts very little range expansion for reef coral assemblages. Compared to a 2010 to 2019 baseline, CERES predicts that new coral reef will amount to less than 25 km^2^ by the end of the century, which is three orders of magnitude lower than the estimated net decline (Fig. 3A). Most of this simulated range expansion occurs in Australia (Fig. 4B and fig. S9), although two ensemble members predict greater expansion in the NW Atlantic, to the north of the Florida Reef Tract (fig. S10). Simulated reef coral expansion in the NW Pacific and SW Indian Ocean is negligible this century (figs. S11 and 12). Range expansion is predicted for nonreef coral communities, particularly in southern Australia (fig. S9). However, as stated above, the largest “expansions” of nonreef coral communities are actually due to coral reef degradation, with reef coral assemblages being replaced by more tolerant nonreef assemblages.

In most ensemble members and regions, coral reaches a minimum by 2080 under the SSP2-4.5 scenario (Fig. 3A). If temperatures and pH are kept stable after 2100, coral cover recovery takes place over centennial timescales, with similar total coral cover to the present achieved by the year 2500. Under SSP1-2.6, coral cover instead reaches a minimum around 2060, whereas coral cover reaches almost zero under SSP5-8.5 by the end of the 21st century. Recovery timescales are similar for all scenarios, although this is expected as we treat all scenarios similarly after 2100 (holding the mean temperature and pH to the 2090 to 2099 mean). Coral cover under SSP5-8.5 does not fully recover within the simulation timespan (by 3500), but post-2100 predictions for this scenario may not be meaningful, as the model does not include any fundamental changes in coral ecology and evolution that might result from a near-total loss of coral reefs.

**Fig. 5.**
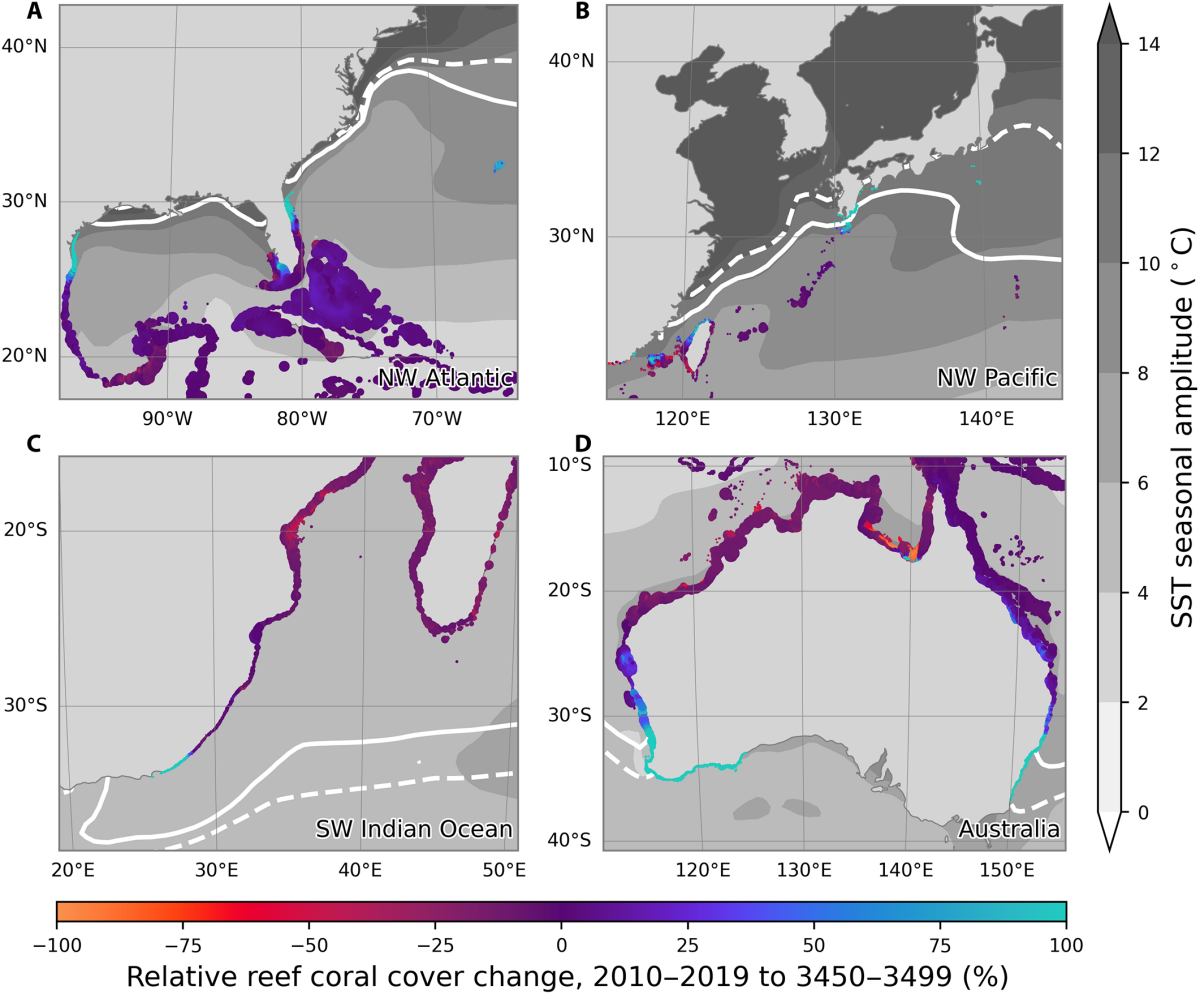
Spatial variability in simulated long-term coral cover change. Marker colors represent the relative change in coral reef cover from 2010-2019 to 3450-3499 under SSP2-4.5 across all ensemble members in CERES for (**A**) NW Atlantic, (**B**) NW Pacific, (**C**) SW Indian Ocean, and (**D**) Australia. Sites are scaled by the absolute reef coral cover between 3450 and 3499. The ocean is shaded by the amplitude of the seasonal cycle of SST, and the solid and dotted white contours represent the annual minimum 18°C isotherm for the preindustrial and post-2090 periods, respectively.

Over centennial timescales, CERES predicts genuine latitudinal range expansion in coral reefs if climate stabilizes (Figs. 3 and 5 and figs. S13 and S14). Most of this range expansion is predicted to occur in the NW Atlantic and Australia (fig. S15), with a potential northward expansion of the Florida Reef Tract, increased coral cover on Bermuda, and considerable southward expansion on the west and east coasts of Australia. CERES also predicts some more minor latitudinal range expansion for coral reefs in Japan and South Africa.

**Fig. 6.**
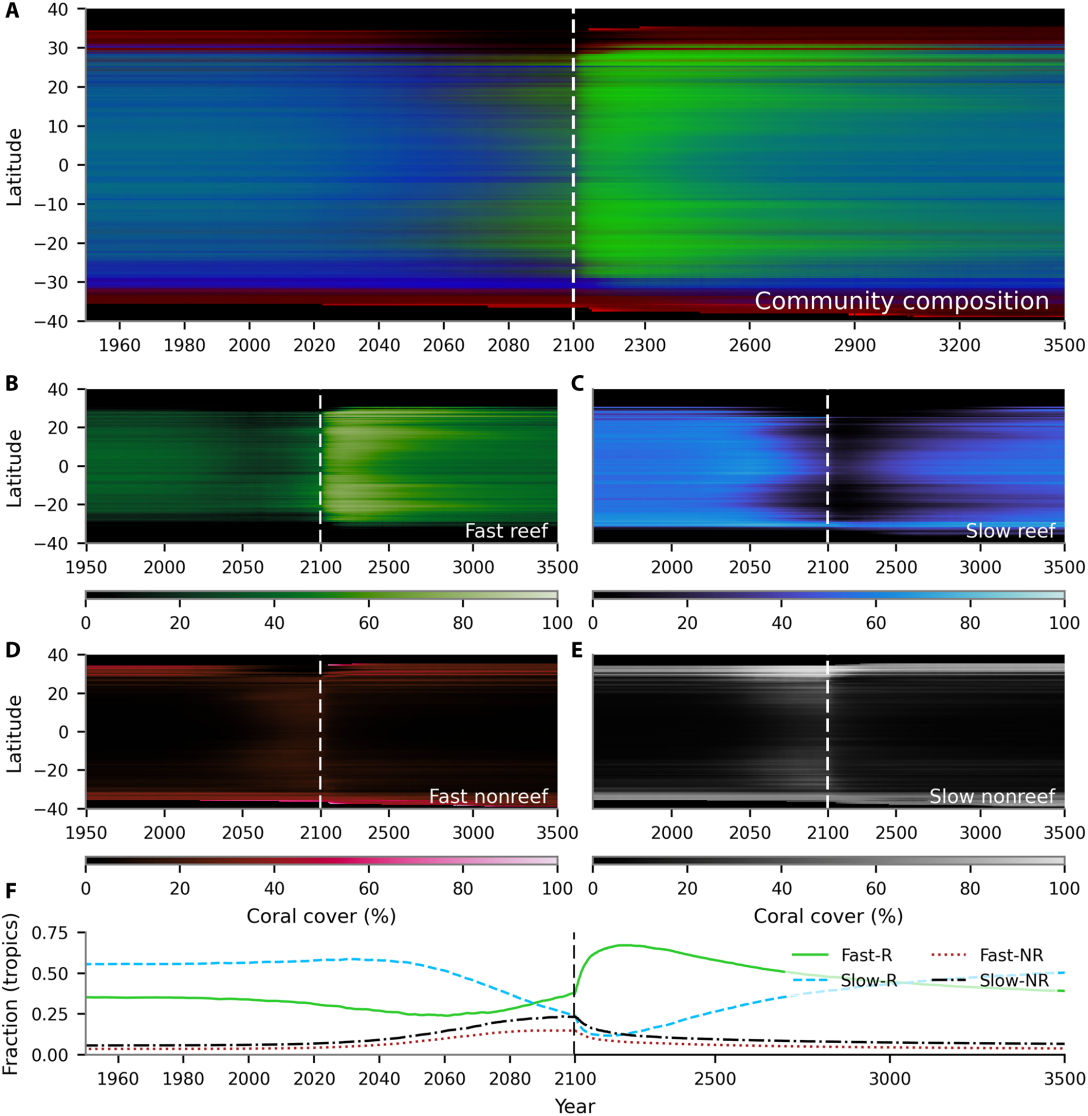
Trends in simulated coral community composition. Colors (**A**) represent the mean community composition per latitude band as a function of time (SSP2-4.5), with fast-growing reef (**B**), slow-growing reef (**C**), and fast-growing nonreef (**D**) proportions broadcast to green, blue, and red channels respectively [there are three degrees of freedom as proportions sum to 1, but slow-growing nonreef proportion (**E**) is effectively represented by brightness]. (**F**) shows the average community composition across the tropics (within 23.5° of the equator).

Last, as well as changes in coral cover, the model predicts changes in community composition between the four assemblages considered in CERES (Fig. 6 and movie S2). At both high and low latitudes, the environmental stress due to 21st-century warming causes the model to predict a general shift away from fast-growing reef corals. Slow-growing (stress-tolerant) reef corals dominate low-latitude assemblages under peak environmental stress, although the model also predicts an increase in nonreef assemblages due to reef degradation. This new community composition peaks after 2050, slightly earlier than the coral cover minimum. As coral cover plateaus and begins to recover, there is a strong rebound in community composition toward fast-growing, competitive corals over a timescale of approximately 100 years. These corals dominate coral reef assemblages during the recovery and colonization phase once warming ceases and continue to dominate community composition for centuries. By the end of the simulation in 3500, community composition is similar to the early 21st century, although with a still elevated proportion of fast-growing corals in the tropics.

## DISCUSSION

### High-latitude coral reefs are ineffective large-scale refugia for corals under anthropogenic climate change

**Fig. 7.**
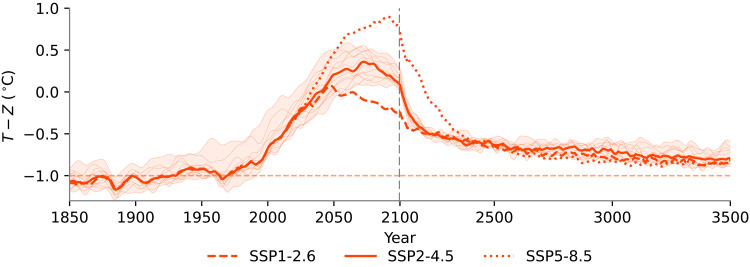
Trends in simulated coral thermal optimum disequilibrium. Plotted lines show the difference between annual mean temperature (*T*) and simulated thermal optima (*Z*) averaged across all reef corals. Thin lines and the shaded area show trajectories for individual models for SSP2-4.5, and the multimodel means for SSP1-2.6 and SSP5-8.5 are respectively shown as dashed and dotted lines. Time series are filtered to remove subdecadal variability. In this model, coral thermal optima equilibrate to 1°C above the annual mean temperature (the horizontal dashed line); values deviating from −1°C therefore represent disequilibrium, i.e., mismatch between coral thermal optima and local temperature.

The geological record shows that coral reef fronts advance and recede in response to long-term climate change (*10*–*13*). CERES simulates range expansions for coral reefs over centennial timescales (Fig. 3), which is fast compared to the temporal resolution of many geological records (*26*). Our simulations are therefore broadly consistent with paleoecological observations. However, our simulations also suggest that the observed and expected rate of ocean warming in the 21st century exceeds the evolutionary adaptive capacity of corals, with a considerable gap emerging between the mean SST and coral thermal optima (Fig. 7). This gap (representing disequilibrium between coral thermal optima and the environment) reaches around 1.5°C in the second half of the 21st century under SSP2-4.5, and re-equilibration occurs, again, over centennial timescales. Therefore, while large-scale range expansion of coral reefs may eventually occur, any new high-latitude reefs will likely emerge centuries too late to act as refugia for the diverse coral assemblages characteristic of modern tropical coral reefs, which face a potentially catastrophic decline within decades.

Our prediction of limited scope for coral reef range expansion this century is complementary to a recent study predicting coral species richness under climate change using environmental niche models (*35*). This study predicted that coral species richness would not increase at high-latitude coral habitats under SSP1-2.6, but could increase in Australia under SSP5-8.5. Together with our results, where a dynamic model suggests that the rate of climate change under SSP5-8.5 far exceeds the capacity of corals to expand their range, it appears unlikely that high-latitude environments will see a meaningful increase in coral species richness over the coming century, regardless of the emissions trajectory.

In CERES, the timescale of long-term range expansion is primarily driven by the coral colony growth rate and genetic variance. Under very high growth rates, reef coral assemblages reach their equilibrium range as early as 100 years after the cessation of warming. Conversely, this expansion requires over 1000 years for very slow colony growth rates (fig. S16). High additive genetic variance, which sets the rate of change of subpopulation thermal optima in response to selective pressure, also drives faster range expansion (fig. S17). In contrast, the rate of range expansion is only sensitive to the effective fecundity, or the rate of population growth due to the new coral colony establishment, under very high values (fig. S18). These findings suggest that the establishment of coral reefs at higher latitudes is limited by the capacity for initially small subpopulations to grow, due to maladaptation to local conditions and greater temperature variability in temperate regions, rather than being limited by larval dispersal. We also suggest that these range expansion timescales most likely represent a lower bound, because CERES assumes that all shallow substrate is habitable for corals, which is clearly not the case.

Observations of major reef-building coral species apparently colonizing high-latitude environments such as Japan have been cited as potential evidence for the capacity of temperate seas to act as large-scale refugia for tropical corals (*17*). While this may be true for certain species, our results support suggestions that increased coral cover in higher-latitude environments in Japan may be driven by local expansions of existing high-latitude coral communities that are distinct from low-latitude coral reefs (*19*). It is therefore unrealistic to expect these environments to harbor coral diversity comparable to low-latitude coral reefs until well beyond 2100, by which time the tropics may have already experienced an irreversible loss in diversity.

Far from acting as refugia for low-latitude corals, higher-latitude coral reefs in the Northern Hemisphere undergo some of the greatest declines in coral cover in our simulations. The simulated decline in coral reefs over the 21st century (for sites beginning with >10% reef coral cover) was reasonably predicted by a linear combination of benthic PAR and the amplitude of the seasonal cycle in SST with a mean absolute error of 9.7% (fig. S19), both of which tend to increase with distance from the equator. It may be unexpected that these environmental variables were more important in determining the simulated coral reef decline than, for instance, the change in temperature or pH (fig. S20). However, the spatial variability in warming and acidification across the tropics and subtropics is relatively low and, in the case of warming, exceeds the rate of evolutionary adaptation everywhere (Fig. 7). Therefore, while ocean warming drives global trends of coral reef degradation, other environmental factors may be key to determining regional and local reef resilience. Because seasonal variability in temperature and benthic PAR tend to respectively increase and decrease with latitude, existing high-latitude coral reefs may experience particularly high compound environmental stress under climate change, exacerbating their simulated decline. This expectation of a greater vulnerability of higher-latitude coral reefs to climate change is supported by another recent modeling study (*36*), although there is limited observational evidence to support this prediction. Our simulations suggest that this latitudinal trend in coral reef vulnerability will not emerge in the Northern Hemisphere until later in the 21st century (fig. S7) and, while this is broadly consistent with global coral cover compilations (*34*), it is inconsistent with recent increases in coral recruitment rates north of 20°N (*14*). We also do not impose an absolute upper thermal limit for coral thermal optima in this model configuration, as coral reefs can adapt to very warm temperatures (*37*); however, introducing an absolute upper thermal limit would result in greater predicted coral reef decline in the tropics, eroding this latitudinal trend in coral reef vulnerability.

Although hyperthermal events from the geological record are not ideal climatic or ecological analogs to the present day, the rate of warming during the Paleocene-Eocene Thermal Maximum (PETM) may have intermittently resembled contemporary warming rates (*26*). Paleontological evidence from the PETM suggests that a long-term expansion toward higher latitudes was preceded by greater extinction risk for high-latitude colonial corals (*38*), which is consistent with our model predictions. Evidence from the PETM also highlights the importance of nonthermal stressors in explaining the response of corals to rapid environmental change (*39*). Conversely, although the PETM severely reduced coral reef formation, the hyperthermal event did not stem the long-term trend of increasing coral diversity throughout the Cenozoic (*38*, *39*). The considerable decline in coral cover predicted by CERES therefore does not necessarily imply a loss of diversity, although caution is required when interpreting these past changes due to the markedly different climatological and ecological context.

### Decoupling between winter temperatures and the coral reef front

In addition to explaining simulated spatial patterns of reef decline over the 21st century, nonthermal stressors also explain much of the variability in the simulated mobility of the latitudinal limits of reef formation, also known as the coral reef front, over centennial timescales and longer (Fig. 5). CERES predicts that the greatest long-term latitudinal expansion of coral reefs will occur in Australia, where the seasonal cycle in temperature is relatively low, allowing reefs to follow the migration of isotherms that set their present-day latitudinal limits. The predicted expansion of the Florida Reef Tract is more moderate, likely limited by the relatively large seasonality in surface temperature.

This temperature variability is even more dramatic in Japan, where the seasonality is greater in amplitude, and the simulated expansion of reef corals is small and largely limited to the northernmost Ryūkyū and Izu islands (Fig. 5). It has previously been suggested that coral range expansion in Japan may be limited by ocean acidification (*40*). CERES simulations with pH fixed to preindustrial levels suggest that acidification is largely responsible for the failure of some coral reefs to fully recover by the year 3500 (fig. S21), because the sensitivity of coral growth rate with respect to pH is fixed in our model. In this scenario, coral reefs appear on the Pacific coast of Kyūshū, but range expansion otherwise remains limited (fig. S22). This suggests that temperature variability may be a more important bottleneck than acidification in throttling the poleward expansion of coral reefs in Japan. We do, however, note that CERES underestimates contemporary coral cover and trends in Japan (figs. S3 and S7), possibly indicating that our model overestimates the impact of seasonal temperature variability on coral population dynamics. Latitudinal light attenuation also limits coral reef range expansion in CERES. In simulations with reduced sensitivity to light, there is greater range expansion in all regions (fig. S23). PAR, while not being the only constraint on the latitudinal limit of coral reefs (*41*, *42*), may therefore still play a first-order role. This is an important consideration, not just in the context of latitudinal light attenuation, but also in relation to anthropogenic sediment inputs due to current and future coastal development or dredging (*43*).

Because CERES has only been assessed against short-term (subcentennial scale) observations, these long-term predictions should be viewed with considerable skepticism. Nevertheless, these simulations support previous assertions that the coral reef front is not solely set by winter SSTs, but rather by a range of environmental parameters (*7*, *37*).

### Disproportionate impact of climate change on acroporid corals may be transient

As with previous studies (*27*, *36*), peak environmental stress during the 21st century disproportionately affects fast-growing reef corals in CERES, the model assemblage that is dominated by acroporid corals (*28*). After individual mass bleaching events, coral community composition in reefs have been widely observed to shift away from (branching) acroporids and toward slow-growing corals with stress-tolerant life-history strategies (*28*, *44*).

CERES predicts that this initial shift in community composition may be rapidly followed by a rebound toward fast-growing reef corals (Fig. 6), which lead to the recovery of low-latitude reefs from the end of the 21st century. Acroporids proliferated during the Pleistocene, possibly due to their rapid growth rate, allowing them to efficiently shift their distribution in response to rapid sea level change (*45*). The proliferation of acroporid corals in response to the availability of new habitat is consistent with our model predictions: Once fast-growing corals (primarily acroporids) are able to adapt to a warmer climate, their population size grows rapidly and allows them to dominate the recently depopulated tropical reefs. This “overcorrection” persists for more than 1000 years in our simulations, more than an order of magnitude longer than the initial decline. On the basis of these simulations, one could hypothesize that (i) the currently observed decadal shifts in coral community composition may be a poor indicator of community composition beyond the mid-21st century, and (ii) the dominant geological signature of past rapid warming may be an increase in the abundance of acroporid corals in fossilized reefs, even if the initial response is the opposite. These are interesting hypotheses but, given the poor constraints on interassemblage competition in CERES, we leave these as open questions.

### Limited scope for 21st-century range expansion despite model uncertainty

CERES reasonably reproduces the large-scale distribution of corals and coral reefs (Fig. 2), as well as observed trends since the late 20st century (fig. S5), and most parameters are constrained by empirical data (see Materials and Methods). However, many parameters are nevertheless subject to considerable uncertainty, and while the model generally performs reasonably against the observational record, the global set of parameters used in our simulations result in discrepancies at a regional scale (e.g., figs. S3 and S7). Here, we demonstrate that the key conclusion of this study—that range expansion alone is unlikely to buffer coral diversity against climate change by the end of the 21st century—is robust with respect to this uncertainty.

The model assumes that all coral colonies follow the same log-normal size distribution, which does not evolve through time. This is a simplifying assumption, as colony size distributions certainly change through space (*46*) and time (*47*). However, the growth rate of coral cover—and, by extension, range expansion—has a relatively low sensitivity to the colony size distribution (see the Supplementary Materials and fig. S24). Along a latitudinal gradient in Australia, higher-latitude sites were found to support larger coral colonies (*46*). If this were to remain true in the future (and in other marginal reef environments), this could further stifle the rate of range expansion.

As previously described, light limitation and acidification contribute to the limited capacity for range expansion among reef corals simulated by CERES. However, although a lower physiological sensitivity to PAR and pH would permit greater range expansion, new reef habitat remains low (≪100 km^2^, considerably lower than the simulated early 21st-century interannual variability) for all values tested (figs. S25 and S26). A broader thermal tolerance and more relaxed heat stress threshold and sensitivity result in delayed coral cover decline and more new reef habitat, but the effects are quite small (figs. S27 to S29).

The additive genetic variance (with respect to the coral thermal optimum) is poorly constrained, although a growing number of studies have found phenotypic variance that translates into additive genetic variance V of order 0.05°C^2^ (*48*, *49*). Greater additive genetic variance reduces the predicted fall in coral cover over the 21st century (fig. S30), due to faster evolution. More coral habitat is generated under both very high and low additive genetic variance, although the latter is likely an artifact of the model initial conditions (the spin-up timescale used in this study may be insufficient for V∼0°C^2^). The most extreme case ( V=0.5°C^2^) increases the potential for new coral cover by an order of magnitude relative to the base case we consider in this study. However, a very high value of V=0.5°C^2^ is inconsistent with most empirical data (*48*, *49*).

The competition parameters in CERES that govern interactions between coral assemblages are among the few parameters that are not explicitly based on empirical data. Values qualitatively similar to those chosen in our simulations are required to reproduce the observations that (i) corals of different life-history strategies coexist at most latitudes (*50*); (ii) coral cover in marginal coral communities is considerably lower than coral cover in reefs; and (iii) reef (high-diversity and reef-forming) coral assemblages dominate over nonreef coral communities at low latitudes. At least within the CERES modeling framework, competition parameters that fulfill the above criteria will tend to produce qualitatively similar (if quantitatively different) behavior to Fig. 6. There were also limited empirical data to constrain the difference in behavior under cold stress between fast-growing and slow-growing assemblages, so we assume that fast-growing assemblages are more sensitive to cold stress (as is the case for heat stress), which is consistent with observations from the Atlantic (*51*). The model is also fairly insensitive to these parameters (figs. S31 to S34). There is some evidence to suggest that acroporid (broadly fast-growing) corals may be more sensitive to changes in pH (*52*), potentially exacerbating the shifts in community composition predicted by CERES. However, this was not incorporated in the present study due to insufficient evidence, and we would not expect them to alter our conclusions given the secondary role acidification plays compared to warming (*8*).

Comparison with observations suggests that CERES may overestimate the sensitivity of coral reefs to seasonal temperature variability (figs. S3 and S7). If this is indeed the case, the particularly dire predictions for reefs in Japan (Fig. 4) may have been overestimated due to strong seasonality in temperature (Fig. 5). However, given that expansion remains limited throughout the 21st century under broader thermal tolerance parameters (fig. S27) and in environments with a weaker seasonal cycle (e.g., fig. S9), it is unlikely that this potential model bias would translate into grossly incorrect predictions for range expansion potential.

Our analyses show that the resilience of reef corals in response to 21st-century warming is sensitive to effective fecundity. Although higher effective fecundity appears less important in setting the rate of coral reef range expansion beyond 2100 (fig. S18), it considerably dampens the effects of 21st-century climate change on coral reefs (fig. S35). Effective fecundity is a complex parameter, translating the existing coral cover into new coral cover generated through the production of larvae, thereby incorporating the actual fecundity (eggs generated per polyp), fertilization rate, larval mortality, settlement likelihood, and survival to sexual maturity. The parameters used in this study represent a best estimate based on values reported in the literature (*28*, *53*, *54*), and considerably higher values of fecundity are inconsistent with the present-day distribution of coral reefs, particularly in the NW Atlantic and Australia (figs. S36 and S37). However, although these parameters may reasonably represent coral assemblages as a whole, it is possible that individual species (with particularly high biological fecundity, and/or very low larval and juvenile mortality) may avoid major decline this century, which is consistent with the observed diverging trajectories of coral reefs in the Caribbean and Indo-Pacific (*55*).

We found no meaningful correlation between any connectivity metric and simulated coral reef trajectories (fig. S38). This contrasts with previous studies that suggested the long-distance dispersal of coral larvae across thermal gradients could, in some cases, improve the capacity of coral reefs to rapidly adapt to ocean warming (*27*, *56*). We hypothesize that the simpler growth rate parameterization and connectivity data, and annual (as opposed to monthly) mean SST considered by previous studies, may have inflated the signal of long-distance connectivity on coral reef trajectories. Our findings suggest that evolutionary processes at a reef scale, perhaps driven by intra-reef connectivity between small-scale thermal regimes (*57*), may play a more important role in shaping the response of coral reefs to short-term environmental change than long-distance connectivity.

Last, CERES does not comprehensively represent all processes that may be relevant to the dynamics of a coral reef, including sea level rise (*58*) and other anthropogenic stressors (*43*), settlement cues and substrate suitability (*59*, *60*), interactions with macroalgae and temperate biota (*7*, *61*), and unresolved scales of oceanographic variability causing our predictions of long-distance connectivity to represent upper bounds (*62*, *63*). However, these unrepresented processes would all generally be expected to further inhibit range expansion, and therefore do not affect the primary conclusions of this study.

In summary, this comprehensive sensitivity analysis demonstrates that it is likely that reefal coral assemblages as a whole will (i) face a major decline this century under realistic emissions scenarios, and (ii) fail to expand their range rapidly enough to buffer diversity against this decline. However, our findings also suggest that certain coral species may avoid catastrophic declines, particularly those with very high effective fecundity, rapid growth rates, and high heritability and phenotypic variance in thermal tolerance.

### Emission cuts and management are critical for the future of coral reefs

Despite high-latitude seas still frequently being referred to as potential refugia, our conclusion to the contrary will be expected for many ecologists, given the abundance of evidence that the functioning of marginal coral communities is fundamentally distinct from tropical reefs (*21*, *22*). What may be more unusual are our pessimistic projections for corals outside the tropics. Although these ecosystems lack the diversity of tropical coral reefs (*64*), they nevertheless provide important ecosystem services, and the proactive implementation of management strategies may be essential to mitigate the impacts of climate change on these unique communities (*21*). In particular, despite the recent success of corals in some high-latitude communities in Japan (*17*), this success may be transient and should not be misinterpreted by policymakers as evidence that management is low priority. Most recent studies exploring the potential of high-latitude environments in Japan to act as refugia for corals emphasized the pressing need for active management to conserve these communities (*18*, *19*).

Although we did not consider anthropogenic stressors such as overfishing and pollution in our simulations, our results suggest that the presence or absence of nonthermal stressors (such as light limitation) that reduce the baseline growth potential for corals can be the difference between tolerable and catastrophic coral cover decline. Coral reefs in Hawai’i exposed to less pollution and greater fishing restrictions have demonstrated greater resilience to thermal stress (*65*), so reducing nonthermal stressors where possible will be increasingly essential for limiting unmitigable damage from heat stress. These factors may also further limit the potential for range expansion; for instance, minimal recent range expansion among corals in Florida relative to historical variability has been attributed to anthropogenic stressors such as sedimentation and terrestrial run-off (*66*).

Last, and perhaps most importantly, our results underline the sensitivity of the future of coral reefs to greenhouse gas emissions trajectories over the 21st century. The geological record demonstrates that a major reduction in coral reef formation is not necessarily equivalent to a reduction in coral diversity (*38*, *39*). Likewise, the proposed inability of high-latitude seas to buffer coral diversity against climate change does not preclude the existence of other refugia, such as microrefugia or mesophotic reefs [although, similarly to the high latitudes, these environments are also home to functionally distinct communities (*22*). Nevertheless, diversity considerations aside, the predicted differences between coral reef futures under SSP1-2.6, SSP2-4.5, and SSP5-8.5 are considerable (Fig. 3), and these differences will have an enormous impact on the communities that depend on healthy coral reefs for their livelihoods (*2*). Our results support a growing body of evidence (*27*, *35*, *36*, *67*) that any reduction in emissions will be highly consequential for coral reefs, even if specific climate targets are missed. These impacts will shape not only coral reef trajectories but also the lives of those who depend on them well beyond the end of the century.

## MATERIALS AND METHODS

### Model overview

CERES simulates eco-evolutionary and range expansion dynamics for shallow-water corals under climate change using a simple metacommunity framework, expanding on earlier studies (*27*, *68*). For a set of i discrete subpopulations and j competing coral groups over k time steps, CERES simulates the response of live coral cover, $C_{\mathrm{ijk}}$ to SST ( $T_{\mathrm{ik}}$ ), pH ( $P_{\mathrm{ik}}$ ), and benthic PAR ( $I_{\mathrm{ik}}$ ) (Table 1). We model a single quantitative trait under selection, the population-mean thermal optimum $z_{\mathrm{ijk}}$ , with additive genetic variance $V_{j}$ . In this model configuration, we consider “reef” corals, representing the high-diversity coral assemblages characteristic of contemporary tropical coral reefs, and “nonreef” corals, representing the low-diversity coral assemblages characteristic of higher-latitude nonreefal coral communities. We emphasize that these groups do not represent any one specific assemblage of coral species, and both groups refer to reef-building (scleractinian) corals. The purpose of these simulations is not to perfectly reproduce the distribution or short-term dynamics of any particular coral species, but rather to represent the processes that dominate the multidecadal response of corals to environmental change in coral reefs and nonreefal coral communities.

**Table 1.** Model variables. Intermediate variables used solely for computation (e.g., $g_{\mathrm{ijk}}$ ) are not listed.

Symbol	Units	Variable
$C_{\mathrm{ijk}}$	–	Fractional coral cover
$z_{\mathrm{ijk}}$	K	Mean thermal optimum trait value
$A_{i}$	m^2^	Habitable area
$T_{\mathrm{ik}}$	K	Temperature
$R_{\mathrm{ik}}$	mol m^−2^ day^−1^	PAR
$P_{\mathrm{ik}}$	–	pH
$t_{k}$	years	Time

Similarly to previous studies (*27*, *36*, *67*), we model two competing life-history strategies for reef corals—fast-growing (Fast-R), and slow-growing (Slow-R)—and likewise for nonreef corals (Fast-NR and Slow-NR). We assume that fast-growing corals have greater maximum growth rates, lower thermal stress thresholds, and greater dispersal potential (*28*). On the basis of the observed latitudinal distribution of tropical coral reefs and nonreefal coral communities, we parameterize reef coral assemblages as having narrower thermal tolerance and stress thresholds, but a competitive advantage over nonreef coral assemblages under optimal conditions (Table 2). We consider anywhere within 20 m depth and within 45° of the equator to be potentially habitable for shallow-water corals and assume that reef can cover at most a certain fraction $r_{f}$ of this area. We set $r_{f}=\frac{1}{8}$ , based on comparison with the Allen Coral Atlas (*33*).

**Table 2.** Model parameters. F-R, fast-growing reef coral; S-R, slow-growing reef coral; F-NR, fast-growing nonreef coral; S-NR, slow-growing nonreef coral. Dispersal kernels AM and GR refer to *A. millepora* and *G. retiformis*, respectively (*92*).

Symbol	Units	Variable	F-R	S-R	F-NR	S-NR
$s_{0,j}$	m year^−1^	Max. linear extension rate	0.05	0.01	0.05	0.01
$w_{j}$	°C	Thermal tolerance	6	6	8	8
$w_{H,j}$	°C	Heat stress tolerance	0.5	1.0	0.5	1.0
$z_{H,j}$	°C	Heat stress (relative) threshold	1.5	1.5	3.5	3.5
$w_{C,j}$	°C	Cold stress tolerance	0.75	1.5	0.75	1.5
$z_{C,j}$	°C	Cold stress (relative) threshold	8	8	10	10
$w_{A,j}$	°C	Evolutionary limit tolerance	6	6	8	8
$z_{A,j}$	°C	Evolutionary limit threshold	24.2	21.3	22.9	19.1
*V*	°C^2^	Additive genetic variance	0.05	0.05	0.05	0.05
$\alpha_{\mathrm{jj}}$	–	Linear competition parameters	–	–	–	–
$f_{j}$	–	Effective fecundity	0.008	0.1	0.008	0.1
–	–	Dispersal kernel	AM	GR	AM	GR
pH^0^	–	Reference pH (at $s_{0}$)	8.1	8.1	8.1	8.1
$c_{\text{pH}}$	–	Extension rate sensitivity to pH	0.5	0.5	0.5	0.5
$I_{\text{sat},j}$	mol m^−2^ day^−1^	Saturation PAR	15	15	15	15
μ	–	Colony size distribution μ	−4.2	−4.2	−4.2	−4.2
σ	–	Colony size distribution σ	1.9	1.9	1.9	1.9
$C_{\text{min}}$	–	Minimum nonzero coral cover	10^−9^	10^−9^	10^−9^	10^−9^
$C_{\text{throttle}}$	–	Selection throttling threshold	10^−3^	10^−3^	10^−3^	10^−3^
$m_{N,j}$	–	Natural mortality	0	0	0	0
$r_{f}$	–	Habitable fraction	0.125	0.125	0.125	0.125

### Experimental design

For each CMIP6 model used, we initialize coral cover from the Allen Coral Atlas (*33*) and the thermal optimum trait value to the 90th percentile SST from the preindustrial control run (piControl). This accelerates model convergence, but the convergent state is insensitive to the initial conditions. We spin up the model for 5000 years by looping the ~500-year piControl forcing, as the model reaches a steady state in terms of coral cover and community composition after approximately 1000 to 4000 years (figs. S39 and S40). This ensures that any response to imposed historical or projected forcing is genuinely a response to a change in forcing, rather than an adjustment from the initial conditions. We then sequentially impose historical (1850 to 2014) and SSP1-2.6, SSP2-4.5, or SSP5-8.5 (2015 to 2099) forcing. For model assessment purposes, we extend the CMIP6 historical run to 2020 using SSP2-4.5 forcing, because this more realistically represents true emissions (*69*). Last, we extend the ScenarioMIP forcing beyond 2100 by computing the difference between piControl and 2090 to 2100 SST and pH, and adding this to piControl forcing. This is not intended to represent a realistic post-2100 climate trajectory (all SSP2-4.5 and most SSP1-2.6 and SSP5-8.5 simulations were not extended beyond 2100 in CMIP6), but does permit us to investigate the long-term equilibrium response of corals to anthropogenic warming.

We assess model performance by comparing (i) modeled reef-forming coral cover averaged across 2010 to 2020 against the Allen Coral Atlas; (ii) modeled coral cover trends from 1978 to 2019 against compilations across GCRMN regions (*34*); (iii) modeled changes in coral cover in Japan against observations of new species occurrences (*17*); and (iv) modeled changes in coral colony density against historical records (*14*).

### Environmental data

At the time of writing, 12 models contributing to CMIP6/ScenarioMIP6 have SST and pH data at monthly temporal resolution, covering piControl, historical, SSP1-2.6, SSP2-4.5, and SSP5-8.5 scenarios, and with a transient climate response within a range deemed reasonable, 1.2° to 2.4°C (*32*). These models are CESM2, CESM2-WACCM, CMCC-ESM2, CNRM-ESM2-1, GFDL-CM4, GFDL-ESM4, IPSL-CM6A-LR, MIROC-ES2L, MPI-ESM1-2-LR, MPI-ESM1-2-HR, NorESM2-LM, and NorESM2-MM (*70*–*78*). We do not screen models based on pH because there is less variability between models (*79*). We use model output on the native grid where possible.

Owing to the higher resolution of the CERES grid versus the ocean grids used in models contributing to CMIP (ranging from c. 20 km for GFDL-CM4, to c. 100 km for MPI-ESM1-2-LR), there is mismatch between the land-sea masks. We therefore interpolate data for land cells in the CMIP models to ensure data coverage for all CERES cells. We then compute the SST and pH for all CERES cells using nearest-neighbor interpolation. Last, we attempt to correct for time-invariant biases in SST and pH using the “delta method” (*80*), i.e., computing the difference between monthly climatological SST and pH from 1986 to 2014 for CMIP fields and observational products, and then adding this (monthly climatological) offset to CMIP fields. We use SST from the 5-km NOAA Coral Reef Watch CoralTemp product (*81*), and pH from the CMEMS Global Ocean Surface Carbon product (*82*).

We use SST rather than benthic water temperature due to the coarse horizontal resolution of the CMIP models used in this study. The average depth of coastal cells used in these GCMs is considerably deeper than the allowed depth for “habitable” cells in CERES, so SST is therefore likely a better approximation for the water temperature experienced by corals.

We obtain benthic PAR from an observational product (*83*), which estimates monthly climatological benthic PAR based on surface irradiance, water-column light attenuation from ocean color, and bathymetry. This product is made available at the same resolution as GEBCO 2023 (i.e., 15 by 15 arc sec). We first interpolate any missing values using the “setmisstodis” distance-weighted interpolation algorithm in “cdo,” and then compute the benthic PAR for each CERES cell as the mean across all benthic PAR values within a water depth of 20 m. We note that, because of the nonlinear (exponential) decay in PAR with depth, computed benthic PAR values based on the mean depth will likely be an overestimate compared to the true mean benthic PAR.

### CERES ecological dynamics

As a reminder, the subscripts i , j , and k respectively refer to subpopulations, coral groups, and time steps. We assume that live coral cover changes through the growth of existing corals through budding, the establishment of new coral colonies through sexual reproduction, and mortality. During months without spawning, the rate of change of coral cover is given by the following differential equation

$$\frac{d}{\mathrm{dt}}C_{\mathrm{ij}}\left(t\right)=\left(g_{\mathrm{ijk}}+\frac{1}{2}V_{j}{\left.\frac{\partial^{2}g}{\partial z^{2}}\right|}_{z_{\mathrm{ijk}}}\right)C_{\mathrm{ijk}}$$ (1)

where $g_{\mathrm{ijk}}$ is the growth rate function (year^−1^), and $\frac{1}{2}V_{j}{\left.\frac{\partial^{2}g}{\partial z^{2}}\right|}_{z_{\mathrm{ijk}}}$ is the genetic load due to variability in z around $z=z_{\mathrm{ijk}}$.

The growth rate function is in turn defined as

$$\begin{array}{c} g_{\mathrm{ijk}}=g_{0,j}\left(1+G_{\text{SST}}+G_{\text{pH}}+G_{I}+G_{C}\right)\\ \;-m_{0}\left(M_{N}+M_{H}+M_{C}+M_{S}\right) \end{array}$$ (2)

where $g_{0,j}$ and $m_{0,j}$ (year^−1^) are respectively the growth and mortality rate coefficients, and G and M terms (unitless) respectively determine the environmental dependence of coral cover growth and mortality. We assume that the linear extension rate of corals scales with the sum of the effects of temperature ( $G_{\text{SST}}$ ), pH ( $G_{\text{pH}}$ ), benthic PAR ( $G_{I}$ ), and competition ( $G_{C}$ ), i.e., there are no interactive effects. We define these functions as follows

$$G_{\text{SST}}=\text{exp}\left[-\frac{{\left(T_{ijk}-z_{ijk}\right)}^{2}}{2w_{j}^{2}}\right]-1$$ (3)

$$G_{\text{pH}}=r_{\text{pH}}\left(P_{\mathrm{ik}}-P_{0,\mathrm{ik}}\right)$$ (4)

$$G_{I}=\text{tanh}\left(\frac{R_{\mathrm{ijk}}}{R_{s,j}}\right)-1$$ (5)

$$G_{C}=-\sum_{f}\alpha_{\mathrm{fj}}C_{\mathrm{ifk}}$$ (6)

The Gaussian form of the temperature function is based on previous coral metapopulation studies (*27*, *56*) and coral thermal performance curves (*84*). We set the reef-forming coral thermal tolerance breadth $w_{j}$ to 6°C, consistent with empirical data (*84*, *85*). Because higher-latitude coral communities can survive a higher amplitude seasonal cycle, we add +2°C to $w_{j}$ for the nonreef corals. We assume that the coral extension rate responds linearly to pH, such that a reduction in 1 pH unit reduces the extension rate by 50% (*52*). Coral calcification rate is widely modeled as a hyperbolic tangent response to PAR (*86*, *87*), with a saturation value of $R_{s}$ . Here, we set $R_{s}=15$ mol m^−2^ day^−1^, a lower-bound estimate from (*87*). Although some short-term empirical studies found a positive growth rate in dark conditions (*87*), in this study, we assume that shallow-water corals cannot grow in complete darkness. We do not consider the potential for photoacclimation, photoinhibition, or shifts in energy sources. Last, we model competition as a linear interaction between coral groups (*27*), set by a competition matrix α , where $\alpha_{R,\text{NR}}=1.2$ , $\alpha_{\text{NR},R}=0.8$ , $\alpha_{R,R}=2$ , and $\alpha_{\text{NR},\text{NR}}=10$ . In other words, when all other parameters are equal, reef (R) corals have a competitive advantage over nonreef (NR) corals. These competition parameters reproduce the observations that (i) differing life-history strategies coexist across the latitudinal range of corals (*50*), and (ii) despite the greater environmental tolerance required to survive at higher latitudes, nonreefal coral communities exist at relatively low abundance at high latitudes, and reef-associated communities dominate in the tropics (*64*). It is possible for $G_{\text{SST}}+G_{\text{pH}}+G_{I}+G_{C}<-1$ , representing compound environmental stress. In this case, the negative value can be interpreted as additional mortality.

To convert coral colony linear extension rates into a coral cover growth rate, we assume that coral colony size structure is log-normal, as is commonly observed in coral reefs (*30*), with shape parameters $\mu_{j}$ and $\sigma_{j}$ . In this case, $g_{0,j}=2\sqrt{\pi }s_{0}c_{0}\text{exp}\left(-\frac{1}{2}\mu_{j}-\frac{3}{8}\sigma_{j}^{2}\right)$ , where $s_{0}$ is the optimal linear extension rate, and $c_{0}=1$ m^−1^ (see the Supplementary Materials). We set $s_{0}$ to 50 and 10 mm year^−1^ for fast-growing and slow-growing corals, the average across their respective life history strategies (*28*). We also set μ=−4.2 and σ=1.9 for all corals, based on the geometric mean across a range of tropical coral species (*30*). To avoid requiring a complex size-structured model, we assume that these shape parameters do not vary, although we acknowledge that this is a model limitation that could be improved in the future (*46*, *47*).

We assume that the mortality rate of corals scales with the sum of the effects of natural background mortality ( $M_{N}$ ), heat stress ( $M_{H}$ ), cold stress ( $M_{L}$ ), and an absolute lower thermal limit ( $M_{A}$ ). We do not impose an absolute upper thermal limit. We define these functions as follows

$$M_{N}=m_{N,j}$$ (7)

$$M_{H}=\frac{{\left[T_{ijk}-\left(z_{ijk}+z_{H,j}\right)\right]}^{2}}{2w_{H,j}^{2}}\delta_{T_{\mathrm{ijk}}>z_{\mathrm{ijk}}+z_{H,j}}$$ (8)

$$M_{L}=\frac{{\left[T_{ijk}-\left(z_{ijk}-z_{C,j}\right)\right]}^{2}}{2w_{C,j}^{2}}\delta_{T_{\mathrm{ijk}}<z_{\mathrm{ijk}}-z_{C,j}}$$ (9)

$$M_{A}=\frac{{\left(T_{\mathrm{ijk}}-z_{A,j}\right)}^{2}}{2w_{A,j}^{2}}\delta_{T_{\mathrm{ijk}}<z_{A,j}}$$ (10)

**Fig. 8.**
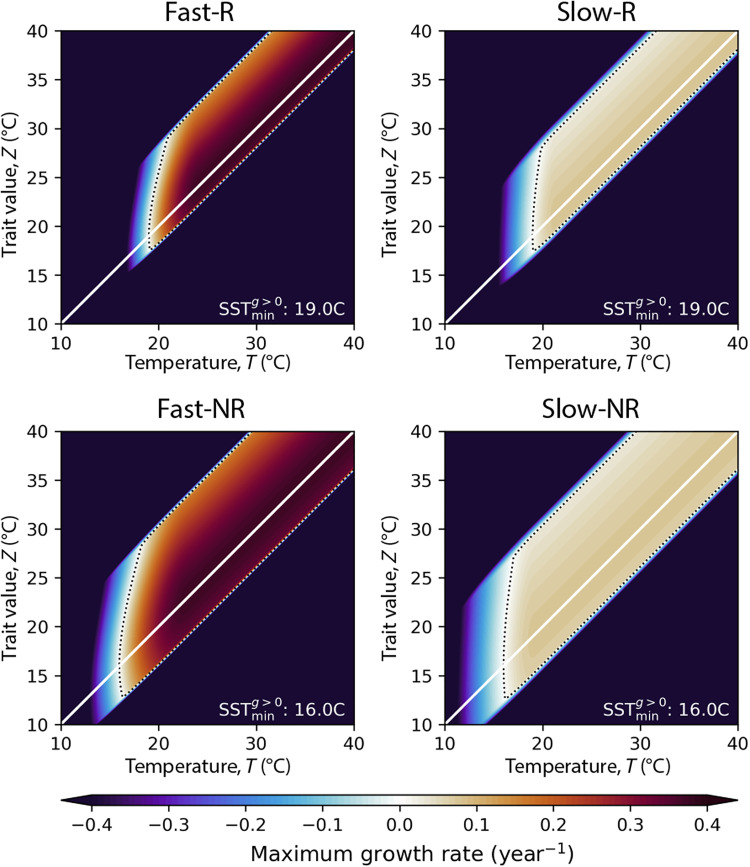
Modeled coral growth rate parameterization Colors represent the optimal population growth rate as a function of the monthly mean temperature (*x* axis) and the thermal optimum trait value *z* (*y* axis). R, reef; NR, nonreef. The dotted black line bounds the region where positive growth is possible, and the white line corresponds to the temperature and thermal optimum being equal. The absence of positive growth rates at low temperatures is due to the absolute limit imposed in the model configuration.

Here, $\delta_{X}=1$ if X is true, and 0 otherwise, such that $M_{N}$ , $M_{H}$ , and $M_{L}$ are only nonzero under stress. We set $m_{0,j}=1$ year^−1^ for correct units (no scaling is required, contrary to the extension rate terms, assuming these mortality rates refer to coral cover). In this study, we set $m_{N,j}=0$ , which assumes that all mortality is due to environmental stress. We also define the temperature difference between the thermal optimum, $z_{\mathrm{ijk}}$ , and the onset of heat stress– and cold stress–induced mortality as $z_{H,j}$ and $z_{C,j}$ , respectively. The heat stress threshold for corals is often set as the mean monthly maximum (MMM) SST + 1°C (*81*), so we set $z_{H,j}=1.5$°C for reef-forming corals because the thermal optimum in our simulations usually equilibrates slightly below the MMM. There are fewer empirical constraints for $z_{c,j}$ , so we set this value to 8°C for reef-forming corals, such that $z_{C,j}+z_{H,j}$ is less than the amplitude of the seasonal cycle at most tropical coral reefs (*37*). As above, higher-latitude coral communities survive a greater range of temperatures than corals in lower-latitude coral reefs, so we add +2°C to both $z_{H,j}$ and $z_{C,j}$ for nonreef corals. The parameters $w_{H,j}$ and $w_{C,j}$ are directly related to the accumulated heat stress required to cause coral mortality (see the Supplementary Materials). Because fast-growing corals such as Acroporidae tend to experience greater bleaching and mortality during marine heatwaves (*44*), we set $w_{H,j}$ to 0.5° and 1.0°C for fast-growing and slow-growing corals, equivalent to mass mortality thresholds of around 9 and 18 degree-heating weeks, respectively (*44*, *88*). Mortality due to cold stress is comparatively poorly constrained, but we set $w_{C,j}$ to 0.75° and 1.5°C for fast-growing and slow-growing corals, respectively, equivalent to conservative mass mortality thresholds of around 13 and 27 degree-cooling weeks (*89*). Last, although we do not explicitly constrain $z_{\mathrm{ijk}}$ , as this trait is free to evolve, we add a growth rate penalty below an absolute lower thermal limit $z_{A,j}$ (*27*), with tolerance $w_{A,j}$ (°C). In the absence of better constraints, we set $w_{A,j}=w_{j}$ , and set $z_{A,j}$ such that the minimum temperature at which growth is possible is 19° and 16°C for reef- and community-type corals, respectively, slightly above the winter temperatures where coral reefs and communities are presently found (*64*). The population growth rate for all four modeled coral groups (under otherwise optimal conditions) as a function of the temperature and trait value is shown in Fig. 8.

During months with spawning (taken to be once per year), we add an additional growth term due to the establishment of new coral colonies. We assume that fecundity is time invariant; that larval settling likelihood is proportional to the available “free space” not occupied by corals and is not affected by chemical cues; that all settling takes place over a “short” time period (such that settling dominates changes in $C_{\mathrm{ij}}$ during a settling event); and that a newly established colony has the size of a single polyp. Under these assumptions, there is an analytical solution for the change in coral cover due to a spawning event (see the Supplementary Materials)

$$\begin{array}{c} \begin{array}{c} C_{\mathrm{ij},k+1}=\frac{I_{\mathrm{ijk}}}{{𝕀}_{\mathrm{ijk}}}\left(1-{\mathbb{C}}_{\mathrm{ik}}\right)\left[1-\text{exp}\left(-{𝕀}_{ik}\right)\right]+C_{\mathrm{ijk}}\\ \;I_{\mathrm{ijk}}=\frac{f_{j}}{A_{i}}\sum_{h}\left(M_{\text{hijk}}C_{\mathrm{hjk}}A_{h}\right)\\ \;{𝕀}_{\mathrm{ik}}=\sum_{j}I_{\mathrm{ijk}}\\ \;{\mathbb{C}}_{\mathrm{ik}}=\sum_{j}C_{\mathrm{ijk}} \end{array} \end{array}$$ (11)

where $f_{j}$ is the effective fecundity (unitless) and $M_{\text{hijk}}$ (also unitless) is the potential connectivity between sites h→i , i.e., the likelihood that a coral j larva generated at site h is transported to site i . We set the effective fecundity to $4\times 10^{-4}$ and $5\times 10^{-3}$ for fast-growing and slow-growing corals, respectively, based on the mean number of eggs generated per polyp for each life history strategy (*28*) and conservative assumptions for the proportion of eggs that translate into sexually mature corals (*53*, *54*) (see the Supplementary Materials). The model assumes that all corals are capable of both sexual and asexual reproduction. The onset of sexual reproduction typically occurs 1 to 10 years postsettling (*90*); neglecting this delay may cause the model to overestimate range expansion.

The potential connectivity matrix is based on the EZfate product (*29*), advected using the 1/12° CMEMS GLORYS12 reanalysis (*91*). The output used in these simulations assume that particles (i.e. coral larvae) are released at the surface of the ocean and are passively advected for 60 days following three-dimensional ocean currents thereafter, recording their location at 2-day intervals. However, other release depths and behaviors are available, and the full dataset is openly available for use in other studies. The proportion of larvae that are alive and competent varies through time (*92*), so we compute the potential connectivity matrix as

$$M_{\text{hijk}}=\frac{\sum_{m}\left(M\prime_{\text{hikm}}p_{\mathrm{jm}}\right)}{\sum_{m}\left(p_{\mathrm{jm}}\right)}$$ (12)

where $M_{\text{hikm}\prime }$ is the raw potential connectivity matrix, and $p_{\mathrm{jm}}$ is the proportion of larvae alive and competent at time $\tau_{m}=\left[2,4\cdots ,60\right]$ days after spawning. In reality, $p_{\mathrm{jm}}$ will vary by species, as well as environmental parameters such as temperature (*93*), but we represent $p_{\mathrm{jm}}$ for the fast-growing and slow-growing groups using parameters for *Acropora millepora* and *Goniastrea retiformis* respectively, to account for the generally lower dispersal distances (and presence of brooding reproductive strategies) within the slow-growing group (*92*). Given the broad number of species and global scope considered in CERES, and the general dominance of high-frequency variability over seasonality in potential connectivity (*94*), we randomly sample potential connectivity matrices across all months (this randomness does not introduce any notable uncertainty, see fig. S41) and assume that potential connectivity (mean state and variability) will not change significantly in the future. Although surface currents are expected to change over the coming century, these changes are small compared to the mean state and variability due to eddies, so this assumption is reasonable (*3*, *95*).

### CERES evolutionary dynamics

We allow only one trait to evolve, namely, the (mean) coral thermal optimum $z_{\mathrm{ijk}}$ (with the subscripts i , j , and k again respectively referring to subpopulations, coral groups, and time steps). In reality, other traits may also change through time. However, we focus here on the thermal optimum, as it is changes in the thermal environment that are expected to drive most future decline in coral cover (*8*). The actual thermal optimum trait value for individual coral colonies is distributed around this mean, with additive genetic variance $V_{j}$ (°C^2^). We set $V_{j}=0.05$°C^2^, the product of the narrow-sense heritability of thermal tolerance in corals, $h^{2}∼0.36$ (*96*) (based on the bleaching susceptibility, an imperfect proxy for thermal tolerance), and the phenotypic variance, $V_{P}∼0.10-0.15$°C^2^ (*48*, *49*) (based on bleaching and mortality thermal thresholds). In CERES, $V_{P}$ is held fixed in space and time, based on observed stability in coral genetic variance in response to major disturbances (*97*). We assume that $z_{\mathrm{ijk}}$ can change through time due to (i) stabilizing selection, and (ii) the introduction of genotypes through immigration.

During months without spawning, $z_{\mathrm{ijk}}$ only changes through stabilizing selection

$$\begin{array}{c} \frac{{\mathrm{dz}}_{\mathrm{ijk}}}{\mathrm{dt}}=q_{\mathrm{ijk}}V_{j}{\frac{\partial g_{\mathrm{ijk}}}{\partial z}|}_{z_{\mathrm{ijk}}}\\ q_{\mathrm{ijk}}=\text{max}\left[0,1-\frac{C_{\text{throttle}}}{\text{max}\left[C_{\text{throttle}},2C_{\mathrm{ijk}}\right]}\right] \end{array}$$ (13)

Here, $C_{\text{throttle}}$ is a coral cover threshold below which selection is throttled (*27*), simulating bottlenecks/founder effects, which we arbitrarily set to 10^−3^. Along with $C_{\text{min}}$ , we are not aware of any empirical constraints for these parameters, so the chosen values are arbitrary. However, the model is insensitive to these parameters over a large range of values (figs. S42 and S43), with results only changing noticeably when $C_{\text{throttle}}$ is very large (greater than 1% coral cover).

During time steps with spawning events, the mean trait value $z_{\mathrm{ijk}}$ can also change through mechanism (ii), immigration. We assume that the mean trait value postimmigration is a simple admixture of the preimmigration mean trait value, and the mean trait value across new immigrants, weighted by coral cover (*27*), i.e.

$$\begin{array}{c} z_{\mathrm{ij},k+1}=\frac{C_{\mathrm{ijk}}z_{\mathrm{ijk}}+{\overset{⌣}{C}}_{\mathrm{ijk}}{\overset{⌣}{z}}_{\mathrm{ijk}}}{C_{\mathrm{ijk}}+{\overset{⌣}{C}}_{\mathrm{ijk}}}\\ \;{\overset{⌣}{z}}_{\mathrm{ijk}}=\frac{\sum_{h}\left(M_{\text{hijk}}C_{\mathrm{hjk}}A_{h}z_{\mathrm{hjk}}\right)}{\sum_{h}\left(M_{\text{hijk}}C_{\mathrm{hjk}}A_{h}\right)} \end{array}$$ (14)

Here, ${\overset{⌣}{z}}_{\mathrm{ijk}}$ is the mean trait value among immigrant larvae, and ${\overset{⌣}{C}}_{\mathrm{ijk}}$ is the change in coral cover due to immigration, i.e., Eq. 11 minus $C_{\mathrm{ijk}}$.

## Supplementary Materials

### The PDF file includes:

Supplementary Text Figs. S1 to S43 Legends for movies S1 and S2 References
